# Rare Plaque Rupture Within a Myocardial Bridge Presenting as Acute Anterior STEMI

**DOI:** 10.1016/j.jaccas.2026.107821

**Published:** 2026-04-04

**Authors:** Samir A. Shah, Sonia R. Samtani

**Affiliations:** a George Washington University School of Medicine and Health Sciences, Washington DC, USA; b Department of Cardiology, MedStar Shah Medical Group, Waldorf, Maryland, USA

**Keywords:** chest pain, congenital heart defect, percutaneous coronary intervention, ultrasound

## Abstract

**Background:**

A myocardial bridge (MB) is a congenital coronary anomaly in which a coronary artery tunnels through myocardium. Plaque formation and rupture typically occur proximal to the bridged segment. Rupture within an MB is rare and presents unique therapeutic challenges.

**Case Summary:**

A 61-year-old male smoker presented with refractory angina pectoris and anterior ST-segment elevations. Emergent percutaneous coronary intervention was pursued, and intravascular ultrasound confirmed plaque rupture within an MB. After balloon angioplasty, a 3.0 × 30 mm drug-eluting stent was deployed at high atmosphere, restoring TIMI flow grade 3 and providing necessary radial strength.

**Discussion:**

This case is atypical, as rupture occurred at the distal portion of the bridge. While guidelines recommend conservative management given risk of stent fracture and restenosis, acute ST-segment elevation myocardial infarction with recurrent pain necessitated intervention. Intravascular ultrasound confirmed diagnosis and guided treatment.

**Take-Home Message:**

Percutaneous coronary intervention can be safely performed in rare cases of MB plaque rupture when clinically imperative.

## Visual Summary

**Figure:**
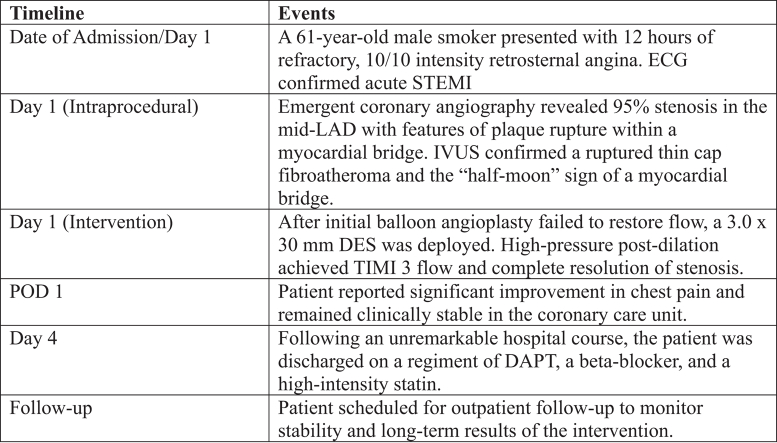

## History of Presentation

A 61-year-old man presented to the emergency department with refractory, retrosternal angina pectoris rated as 10 out of 10 in intensity. The symptoms began abruptly and persisted for 12 hours prior to evaluation. The patient denied associated dyspnea, palpitations, diaphoresis, emesis, or prior episodes of chest discomfort. Upon arrival, he was alert and oriented, with a heart rate of 56 beats/min, respiratory rate of 10 breaths/min, and blood pressure of 135/95 mm Hg. Physical examination revealed no cardiac gallops, murmurs, or signs of congestive heart failure.

**Take-Home Messages**

• Plaque rupture associated with a myocardial bridge is a rare but important cause of acute myocardial infarction, and intravascular imaging helps accurately localize lesions and guide management in complex coronary anatomy.
• In selected acute STEMI cases where conservative therapy is not feasible, PCI may be performed despite risks, but further randomized trials are needed to determine the optimal long-term postintervention medical therapy.

## Past Medical History

The patient reported no prior history of atherosclerotic cardiovascular disease, hypertension, diabetes, or dyslipidemia. He had a significant tobacco history of 20 pack-years. He had no prior cardiological evaluation and was not on medical treatment.

## Differential Diagnosis

In a patient presenting with acute retrosternal chest pain refractory to rest and nitrates, the primary diagnostic consideration is acute coronary syndrome, most likely secondary to an acute plaque rupture causing near-total occlusion of a coronary artery. Other considerations include coronary vasospasm, spontaneous coronary artery dissection, and less likely, an aortic dissection involving the coronary ostia. The patient's age, smoking history, and symptoms supported acute coronary thrombosis as the most likely etiology.

## Investigations

Electrocardiography showed 2-mm ST-segment elevations in leads V_1_ through V_4_, consistent with an acute anterior ST-segment elevation myocardial infarction (STEMI).

## Management

**Figure 1:**
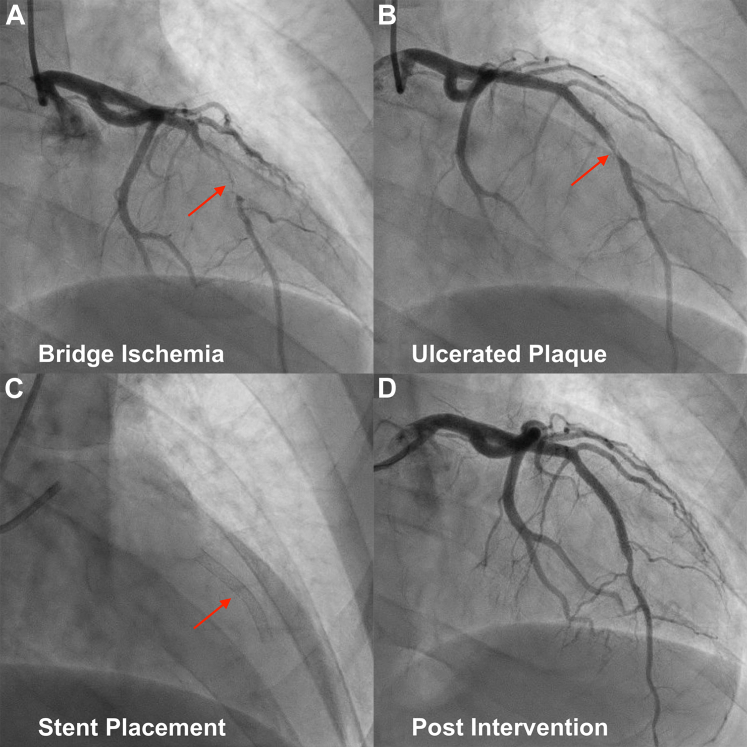
Coronary Angiography and Intervention in Acute Anterior STEMI With Myocardial Bridge (A) Severe systolic compression of the mid-LAD consistent with bridge ischemia (arrow). (B) Evidence of ulcerated plaque within the bridged segment (arrow). (C) Placement of drug-eluting stent across the lesion (arrow). (D) Final angiography demonstrating resolution of the stenosis and restoration of TIMI flow grade 3 without dissection or perforation. LAD = left anterior descending artery; STEMI = ST-segment elevation myocardial infarction.

After a loading dose of 324 mg of aspirin from emergency medical services en route to the hospital, the patient received a 180-mg tablet of ticagrelor and a 5,000-U unfractionated heparin bolus. Emergency coronary angiography was performed, and percutaneous access was obtained via the right radial artery with a 6-F sheath using the Seldinger technique. Diagnostic angiography demonstrated 95% stenosis in the mid left anterior descending artery (LAD) (Figure 1A) with angiographic features suggestive of plaque rupture at the site of a myocardial bridge (MB) (Figure 1B). Initial balloon angioplasty using a 2.0 × 15 mm balloon failed to restore luminal patency. Intravascular ultrasound (IVUS) was employed to characterize the lesion morphology, demonstrating the typical characteristics of an MB, including “half-moon” sign and arterial compression (Video 1). Additionally, ruptured thin cap fibroatheroma was confirmed within the bridged segment. To counteract the dynamic systolic compression and stabilize the culprit lesion, a 3.0 × 30 mm drug-eluting stent was deployed (Figure 1C). High-pressure postdilation was performed using a 2.0 × 15 mm compliant balloon to ensure adequate stent apposition.

Final angiography demonstrated complete resolution of the stenosis, restoration of TIMI flow grade 3, and no evidence of edge dissection or perforation (Figure 1D). The patient remained stable throughout the procedure.

Postintervention management included continuation of anticoagulation during the procedure, initiation of high-intensity statin therapy, beta-blocker administration, and continuation of dual antiplatelet therapy with aspirin and ticagrelor.

## Outcome and Follow-Up

The patient was admitted to the coronary care unit after the intervention. On the first day after catheterization, he reported significant improvement in chest pain and had an unremarkable hospital course. He was continued on medical therapy for coronary artery disease with dual antiplatelet therapy, beta-blocker, and statin. After confirming medical stability, the patient was discharged with outpatient follow-up.

## Discussion

Myocardial bridging refers to the intramyocardial course of a major epicardial artery, most frequently the LAD. While often a benign incidental finding, an MB can manifest as stable angina or acute coronary syndrome. Typically, the tunneled segment is spared from atherosclerosis owing to high wall shear stress and dynamic compression, with plaque formation typically developing proximal to the bridged segment because of turbulent flow and retrograde flow waves.

This case is highly atypical, as IVUS confirmed a ruptured plaque located within the bridged segment of the LAD, a phenomenon rarely reported in clinical literature. The presence of both plaque rupture and MB in the same anatomical location created a diagnostic and therapeutic challenge. Previous literature suggests that percutaneous coronary intervention (PCI) within a bridged segment should be avoided when possible, given the risks of stent fracture, restenosis, and continued dynamic compression.^1^^,^^2^ In the setting of acute STEMI, conservative therapy was not a viable option, and the clinical team proceeded with emergent intervention.

Alternative strategies, such as drug-coated balloons with prolonged inflation, have been proposed to treat MB lesions while avoiding permanent scaffolding.^3^ However, drug-coated balloons are currently not approved for de novo acute lesions, and in this instance, the degree of thrombotic burden and vessel recoil observed postdilation required the mechanical support of a drug-eluting stent with its radial strength.

After the intervention, the patient was started on a beta-blocker as first-line therapy, per American College of Cardiology guidelines, to reduce the systolic compression forces on the bridged segment.^4^^,^^5^ While non-dihydropyridine calcium-channel blockers are often considered a second-line alternative for their potential vasospastic protection, there is currently a lack of randomized trial data to define the superior agent in post-PCI management of MBs. Given the absence of contraindications, the standard beta-blocker approach was maintained, though further research is required to optimize therapy in this rare cohort.

In this case, imaging-guided PCI was successful and well tolerated, with no procedural complications and complete resolution of flow-limiting stenosis. This case reinforces the importance of intravascular imaging in identifying atypical lesion locations and planning appropriate intervention.

## Conclusions

This case highlights an unusual but clinically significant presentation of acute anterior STEMI due to plaque rupture within a myocardial bridge. IVUS was critical in establishing the lesion's location and guiding successful PCI. This case demonstrates that PCI can be performed safely and effectively within bridged segments when clinically necessary.

## Funding Support and Author Disclosures

The authors have reported that they have no relationships relevant to the contents of this paper to disclose.

## Appendix

Video 1 Intravascular Ultrasound Characterization of a Myocardial Bridge IVUS imaging of the mid-LAD demonstrates the classic “half-moon” echolucent sign (arrow) consistent with a myocardial bridge, along with dynamic arterial compression of the tunneled segment. These findings confirm the intramyocardial course of the vessel.
